# Hyaluronic acid modified indocyanine green nanoparticles: a novel targeted strategy for NIR-II fluorescence lymphatic imaging

**DOI:** 10.3389/fchem.2024.1435627

**Published:** 2024-07-03

**Authors:** Haiyan Zhang, Xinyu Wang, Yundong Zhang, Jinli Ma, Shaolong Qi, Jianshi Du, Chunxiang Jin

**Affiliations:** ^1^ Department of Ultrasound, China–Japan Union Hospital of Jilin University, Changchun, China; ^2^ Key Laboratory and Engineering Laboratory of Lymphatic Surgery Jilin Province, China-Japan Union Hospital of Jilin University, Changchun, China; ^3^ Key Laboratory of Bioorganic Phosphorus Chemistry and Chemical Biology, Department of Chemistry, Tsinghua University, Beijing, China

**Keywords:** fluorescence imaging, NIR-II imaging, hyaluronic acid, indocyanine green, LYVE-1, lymphatic imaging

## Abstract

The lymphatic system, alongside blood circulation, is crucial for maintaining bodily equilibrium and immune surveillance. Despite its importance, lymphatic imaging techniques lag behind those for blood circulation. Fluorescence imaging, particularly in the near-infrared-II (NIR-II) region, offers promising capabilities with centimeter-scale tissue penetration and micron-scale spatial resolution, sparking interest in visualizing the lymphatic system. Although indocyanine green (ICG) has been approved by the Food and Drug Administration (FDA) for use as a near-infrared-I (NIR-I) region fluorescent dye, its limitations include shallow penetration depth and low signal-to-noise ratio. Research suggests that ICG’s fluorescence emission tail in the second near-infrared window holds potential for high-quality NIR-II imaging. However, challenges like short circulation half-life and concentration-dependent aggregation hinder its wider application. Here we developed HA@ICG nanoparticles (NPs), a superior ICG-based NIR-II fluorescent probe with excellent biocompatibility, prolonging *in vivo* imaging, and enhancing photostability compared to ICG alone. Leveraging LYVE-1, a prominent lymphatic endothelial cell receptor that binds specifically to hyaluronic acid (HA), our nanoprobes exhibit exceptional performance in targeting lymphatic system imaging. Moreover, our findings demonstrate the capability of HA@ICG NPs for capillary imaging, offering a means to assess local microcirculatory blood supply. These compelling results underscore the promising potential of HA@ICG NPs for achieving high-resolution bioimaging of nanomedicines in the NIR-II window.

## 1 Introduction

The lymphatic system plays a vital role in maintaining environmental homeostasis and immune surveillance, making its dysfunction consequential for multiple organ systems. However, the colorless and transparent nature, coupled with the small diameters of lymphatic vessels, pose formidable challenges for accurate identification, even under microscopic examination (Proulx et al., 2013; Hu et al., 2024). Therefore, visualizing the lymphatic system is imperative for studying lymphatic-related diseases. Traditional imaging modalities such as X-rays, computed tomography (CT) scans, and magnetic resonance images (MRIs) have been instrumental in diagnosing and treating lymphatic disorders. Yet, these techniques often fall short in depicting the intricate branching and dynamic changes of lymphatic vessels. In contrast, fluorescence imaging technology, leveraging fluorescent dyes, offers a clear visualization of lymphatic structures and nodes, facilitating real-time monitoring of lymphatic function (Qi et al., 2022).

Fluorescence imaging offers several advantages, including non-invasiveness, high resolution, and real-time capability, making it indispensable for lymphatic system imaging. However, limitations such as biological spontaneous fluorescence and light scattering have impeded the clinical translation of the near-infrared-I (NIR-I) region (650–950 nm). Extending the fluorescence wavelength to the NIR-II region (1,000–1,700 nm) reduces spontaneous fluorescence and tissue background scattering, enabling greater tissue penetration and higher spatial resolution compared to NIR-I window (Hu et al., 2019; Cao et al., 2020; Wang et al., 2023). Consequently, various types of NIR-II fluorescent probes, such as quantum dots and carbon nanotubes, are actively utilized for preclinical vascular imaging and lymphatic imaging (Zebibula et al., 2017; Zhang et al., 2018; Wu C. et al., 2020; Zhang et al., 2020; Chen et al., 2021; Zhang et al., 2022).

Despite their potential, NIR-II probes face delays in clinical application due to biosafety concerns. To address this, the development of NIR-II nanoprobes based on indocyanine green (ICG), a commonly used FDA-approved NIR-I fluorescent dye, is crucial for lymphatic system imaging during this transition period. While the fluorescence emission of ICG in the NIR-II window meets imaging requirements, challenges such as its short half-life, aggregation-caused quenching (ACQ) due to π-π stacking, photobleaching, and lack of lymphatic system targeting constrain its effectiveness in NIR-II lymphatic imaging (Carr et al., 2018). Integrating ICG with nanotechnology has been explored to address these issues, yet deficiencies persist in lymphatic system targeting and persistent imaging capabilities (Wu et al., 2016; Crawford et al., 2020; Wu D. et al., 2020; Wang et al., 2022; Lin et al., 2024). LYVE-1, a type I integral membrane glycoprotein, acts as a receptor for both soluble and immobilized HA. It facilitates lymphatic hyaluronan transport and may play a role in tumor metastasis (Jackson et al., 2001; Prevo et al., 2001; Yang et al., 2017). The crucial role played by HA in lymphatic system targeting transport positions it as an ideal nano-material for lymphatic targeting modification with excellent biocompatible properties.

**FIGURE 1:**
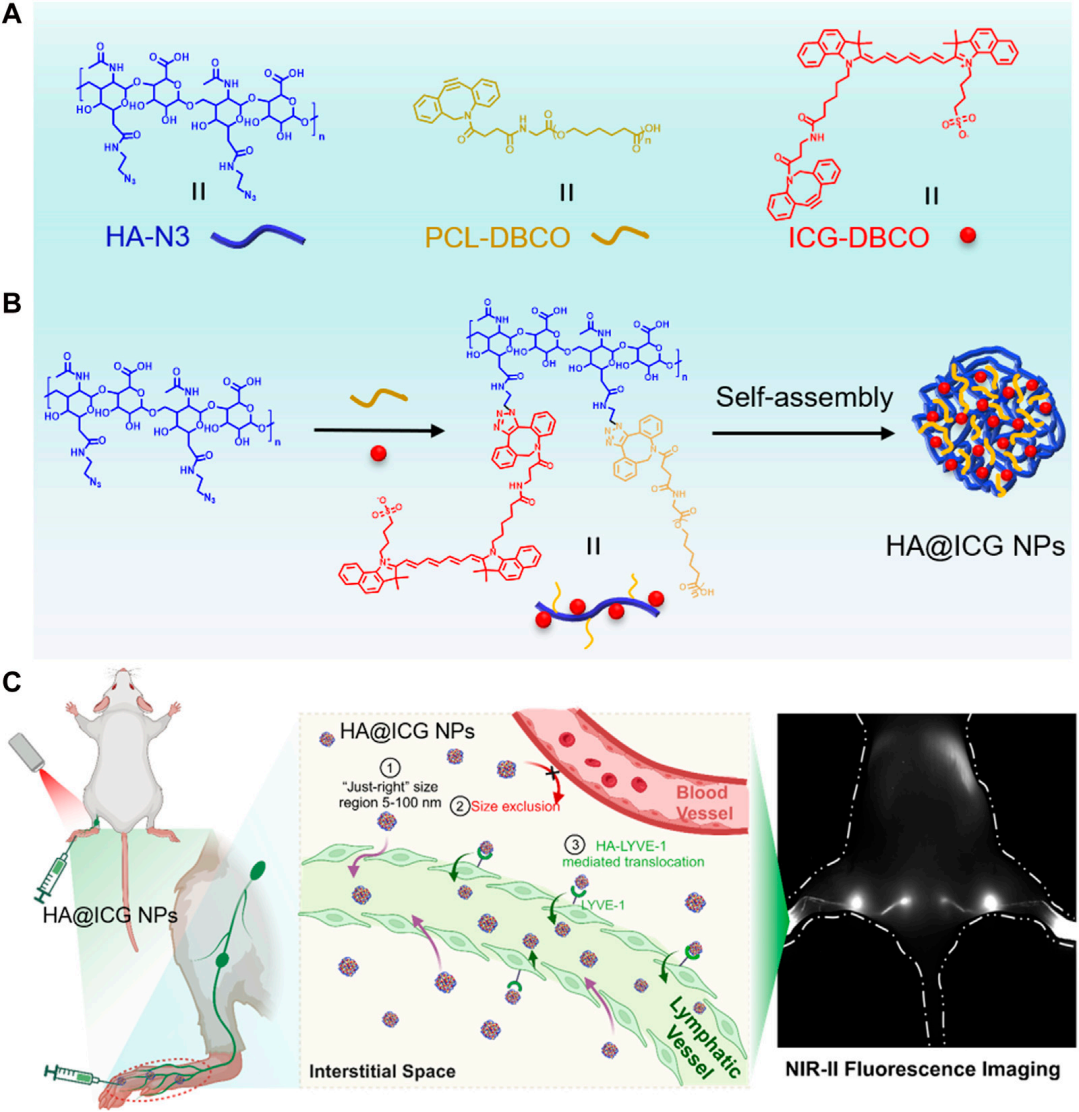
**(A,B)** Illustration of the preparation process for HA@ICG NPs; **(C)** Path of the nanoparticles from the interstitial space into the lymphatic vessels:①② “Size effect”: Nanoparticles of just 5–100 nm in size are excluded from the capillaries and diffuse into the lymphatic lumen via the endothelial space of the lymphatic vessels; ③HA-LYVE-1:Translocation of HA@ICG NPs into lymphatic vessels by the high specific affinity of HA for LYVE-1.

Herein, a biocompatible NIR-II probe was developed by conjugating indocyanine green (ICG) with polycaprolactone (PCL) molecules onto hyaluronic acid (HA) molecules using a copper-free catalyzed click chemical reaction (Figures 1A, B). This unique construction method maximally disperses ICG molecules, significantly reducing fluorescence quenching and self-aggregation caused by π-π stacking interactions. As shown in Figure 1C, HA@ICG NPs have an ideal size for lymphatic drainage, approximately 50 nm, enabling specific entry into lymphatic vessels via the “size effect” and targeting lymphatic vessels through receptor-ligand interactions (HA-LYVE-1) (Swartz, 2001; Trevaskis et al., 2015; Jackson, 2019; Qi et al., 2022). Benefiting from these design principles, this study confirms the excellent biosafety, stability, and extended lymphatic system imaging time window of HA@ICG NPs. By enabling NIR-II imaging of lymphatic vessels, these nanoparticles aid in understanding the patency of target lymphatics. With these attributes, HA@ICG NPs demonstrate significant potential for successful clinical translation.

## 2 Experimental section

### 2.1 Materials and methods

Hyaluronic acid (HA, 10000), DBCO-NHS, PCL-NH_2_, dimethylsulfoxide (DMSO), triethylamine, 2-bromoacetonitrile, acetone, 2-Bromoethyl isocyanate, sodium azide (NaN_3_), ICG-dibenzocyclooctyne (ICG-DBCO), diethyl ether were purchased from Sigma-Aldrich. MTT cytotoxicity assay kits for cell viability detection were bought from Thermo Fisher Scientific. Hoechst was purchased from Beyotime Biotechnology (Shanghai, China). The ICG-dibenzocyclooctyne (ICG-DBCO) was purchased from Xi’an Qiyue Biotechnology Co., Ltd., (Xian, China). Transmission electron microscopy (TEM) investigations were carried out on an Hitachi HT-7650B. Fluorescence spectra was obtained by a Hitachi F-7000 fluorescence spectrophotometer. FTIR spectra was obtained by Fourier transform infrared spectrometer (Horiba Bruker, Germany). Dynamic Light Scattering (DLS) was used to determine the size and size distribution of the nanoparticles using a Zetasizer Nano ZS90 Malvern Instruments. The fluorescence images were taken using a Nikon Fluorescent Inverted microscope Eclipse TS2-FL. All NIR-II images were gathered by InGaAs camera platform.

### 2.2 Preparation of HA@ICG NPs

PCL-NH_2_ (1 g, 500 µmol), DBCO-NHS (200 mg, 500 µmol), and two drops of triethylamine were dissolved in 20 mL DMSO, and the solution was stirring at room temperature for overnight. After the reaction, transferred the reaction mixture into 200 mL of ice-cold diethyl ether, and then centrifuged to remove the supernatant ether solution. The precipitates were washed with acetone three times, centrifuged, and dried to obtain white solid PCL-DBCO (0.92 g, yield: 83.6%).

Dissolve 0.71 g (71 µmol) of HA in 1 mL of pure water, followed by the addition of 20 mL of DMSO. Evaporate the mixture to remove water completely. Once the water is fully removed, add 0.28 g of 2-Bromoethyl isocyanate to the solution and let it react overnight. Upon completion of the reaction, transfer the mixture into 200 mL of ice-cold diethyl ether, and then centrifuge to remove the supernatant ether solution, obtaining a milky transparent liquid. Add this liquid to 200 mL of acetone to precipitate a white precipitate, wash with acetone three times, centrifuge, and dry to obtain white solid HA-Br (0.79 g, yield 80%).

HA-Br (1 g, 79 μmol) was dissolved in 5 mL pure water, stirring thoroughly until completely dissolved, and then add 25 mL DMSO, evaporate the mixture to remove water. NaN_3_ (0.1 g, 1.58 mmol) was dissolved in 1 mL pure water and dropwise into the reaction mixture. The solution was heated slowly under stirring at 80°C for overnight. After the reaction, transfer it to a dialysis bag and dialyze for 8 h. Concentrate by rotary evaporation and precipitate with acetone. Wash the resulting white precipitate three times with acetone, then centrifuge and dry to obtain HA-N_3_ (0.86 g, yield: 87%).

HA-N_3_ (1 g, 80 μmol) was dissolved in 500 μL pure water, and add 3 mL DMSO, evaporate the mixture to remove water. ICG-DBCO (316 mg, 320 µmol), PCL-DBCO (736 mg, 320 µmol) was dissolved in 1 mL DMSO, and dropwise into the above reaction system, and the reaction was stirred for 12 h under room temperature. After the reaction complete, precipitate with acetone. Wash the resulting green precipitate three times with acetone, then centrifuge and dry to obtain HA-PCL-ICG (1.55 g, yield: 77.5%).

Dissolve 6 mg of HA-PCL-ICG in 2 mL of DMSO, and slowly add 10 mL of deionized water with stirring. After dialysis using a dialysis bag (with a molecular weight cutoff of 3,500) for 24 h, the mixture is freeze-dried to obtain HA@ICG NPs.

### 2.3 Cell culture

Human lymphatic endothelial cells (HLECs) were cultured in Endothelial Cell Medium (ECM) containing 5% fetal bovine serum (FBS) and 1% Endothelial Cell Growth Supplement (ECGS) and 1% penicillin-streptomycin solution. Cells were cultured at 37°C in a 5% CO_2_ atmosphere. Cells were harvested from the cell culture medium by incubating in a trypsin solution for 2 min. A 2.0 mL portion of serum-supplemented Endothelial Cell Medium was added to neutralize any residual trypsin. The cells were centrifuged, and the supernatant was discarded. The cells were resuspended in serum-supplemented medium.

### 2.4 Cytotoxicity assessment

Human lymphatic endothelial cells (HLECs) were seeded in 96-well plates at a density of 1.0 × 10^4^ cells per well for MTT assay to evaluate cytotoxicity of HA@ICG NPs. Cultured over the night, cells were treated with the samples at different concentration for 24 h. The media was removed and washed with PBS three times, then the cells were incubated in 20 μL of an MTT solution and 180 μL of media for an additional 4 h of incubation at 37°C. After the MTT solution was removed, 150 μL DMSO was added to each well. The purple crystal was fully dissolved by shaking the table at low speed for 10 min. The absorbance was measured at 490 nm using a microplate reader. Untreated cells in media were used as a control. All experiments were carried out with four replicates.

### 2.5 Cellular uptake of HA@ICG NPs

HLECs were cultured and seeded at a density of 1 × 10^5^ cells per well in 96-well plates for 12 h. HA+HA@ICG NPs Group was pre-treated with 10 mg/mL of HA for 1 h. Subsequently, different components were added and co-incubated with the cells for 2 h and 6 h, respectively. After removal of the culture medium, the dishes were gently rinsed three times with PBS at set time intervals. The cell nuclei were stained with Hoechst 33342 for 10 min. The cells were washed with PBS three times lightly. Finally, the images were taken using a fluorescence microscope.

### 2.6 Animals

All animal studies in this experiment were carried out in accordance with the guidelines and practices outlined in the Guidelines for Animal Care and Use Organizations, the protocol approved by the First Hospital of Jilin University’s Experimental Animal Ethics Committee (Agreement No. 20210642), and Jilin University’s policies on animal experimentation. BALB/c mice (6–8 weeks of age, female) were bought from Liaoning Changsheng Biological Co., LTD. The mice were fed in separate cages with an independent ventilation cage system (IVC) and kept in isolation barriers with continuous feed humidity. After 7 days of adaptive feeding, the mice were divided at random while the indoor temperature was kept at 20°C ± 2°C. Light and darkness were also maintained for 12 h.

### 2.7 NIR-II bioimaging *in vitro* and *in vivo*

The mice were anesthetized with isoflurane and then injected with an imaging agent via the tail vein or subcutaneously. On a two-dimensional InGaAs camera platform from Princeton Instruments and Raptor Photonics, all NIR-II images were gathered. An 808 nm laser with a power density of approximately 78 mW/cm^2^ was used as the excitation laser. To identify the ideal imaging window *in vivo*, emission was captured using several long-pass filters. Then, under 1,000 nm long pass filter, NIR-II biological imaging was seen *in vitro* and *in vivo*.

### 2.8 Long-term biosafety analysis

Each formulation was injected at a concentration of 300 μM, and blood was collected on days 3 and 7. The levels of key functional indicators, including WBC, RBC, Lym, ALT, AST, ALP, BUN, CREA, and so on, were measured in each group according to the instructions. The mice were sacrificed on days 3 and 7 after injection, and their major organs were removed and fixed with an EDTA/formalin solution. All organs were processed according to the routine H&E staining procedure.

### 2.9 Statistical analysis

Data are presented as the mean ± standard deviation. Statistical analysis of data was performed with one-way or two-way analysis of variance (ANOVA). The level of significance was defined as *p < 0.05, **p < 0.01, ***p < 0.001.

## 3 Results and discussion

### 3.1 Preparation of HA@ICG NPs

**FIGURE 2:**
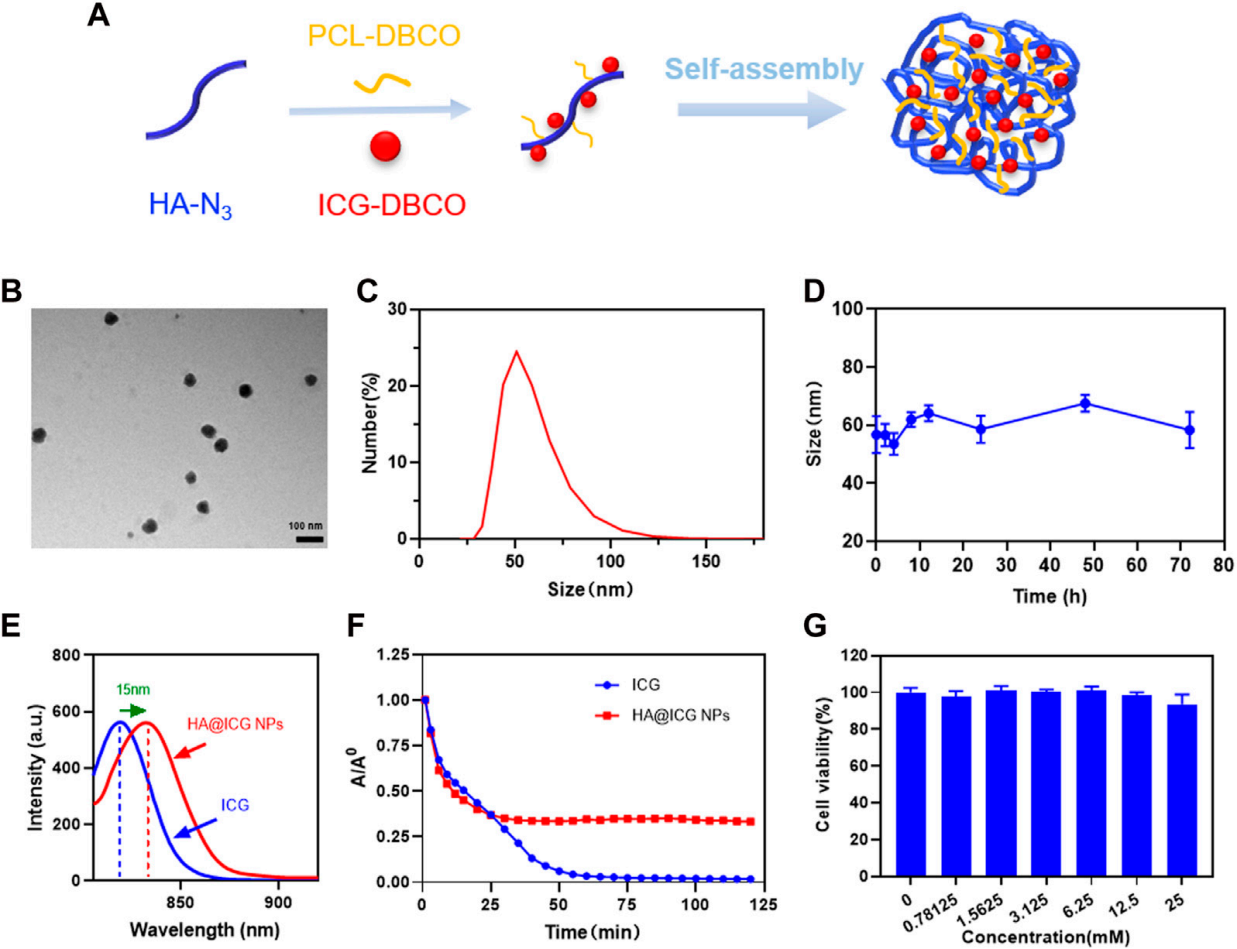
Preparation and characterization of HA@ICG NPs. **(A)** Schematic illustration of the preparation process for HA@ICG NPs. **(B)** TEM images of HA@ICG NPs (Scale bar = 100 nm). **(C,D)** Size distribution and the particle size stability of HA@ICG NPs in PBS over 72 h **(E)** The fluorescence spectra of ICG and HA@ICG NPs. **(F)** Fluorescence changes of ICG and HA@ICG NPs under continuous laser radiation (808 nm, 78 mW cm^−2^) over a period of time in a long-pass filter above 1,000 nm. **(G)** Cell viability of HLECs treated with different concentrations of HA@ICG NPs (*n* = 4).

To reduce fluorescence quenching and self-aggregation caused by π-π stacking interactions, a grafting reaction was employed to conjugate ICG with PCL molecules onto HA molecules, utilizing a copper-free catalyzed click chemistry reaction. Eventually, HA@ICG NPs were successfully synthesized by self-assembly (Figure 2A; Supplementary Figures S1–S3) and fully characterized. In order to filter out the optimal ICG-DBCO/PCL yield ratio, we recorded the emission spectra of ICG and various HA@ICG NPs with different ICG-DBCO to PCL ratios, as shown in Supplementary Figure S4. It demonstrated that ICG exhibited a pronounced emission peak at 815 nm when excited at 780 nm, while HA@ICG NPs show a distinct emission peak at 830 nm, confirming the successful incorporation of ICG-DBCO. Notably, the fluorescence intensity of these nanoparticles diminished with higher ICG-DBCO inputs, potentially due to increased π-π stacking and local concentration, which may lead to fluorescence quenching. Consequently, we determined a 1:1 ratio to be the most optimal yield ratio.

The particle size, as determined by dynamic light scattering (DLS), is approximately 60 nm. According to TEM results, HA@ICG NPs have a spherical structure with an average particle size of 47.20 nm, which essentially agreed with the DLS findings (Figures 2B, C). The literature indicates that nanoparticles that are less than 5 nm in size can readily permeate into the circulatory system from the interstitial space (Zbyszynski et al., 2019). However, the interstitial water channels, which are approximately 100 nm in diameter, are the primary route for chemical transfer from the injection site through the interstitium. Nanoparticles with diameters greater than 100 nm are more likely to persist in the interstitial space. On the other hand, nanoparticles ranging from 5 to 100 nm in diameters appear to easily penetrate lymphatic channels for imaging applications (Ryan et al., 2014; Thomas and Schudel, 2015). The size of the HA@ICG nanoparticles created in this work is ideal for imaging lymphatic channels. Furthermore, the data presented by DLS indicates that particle size variations within a 72-h period in a PBS solution can be disregarded (Figure 2D). We also adopted the cell culture medium containing 10% fetal bovine serum to verify *in vivo* stability of HA@ICG nanoparticles. The results showed no significant changes of hydrodynamic diameter in 48 h (Supplementary Figure S5). These findings verified that the HA@ICG NPs exhibited a high level of physiological stability. The Zeta potential of HA@ICG NPs is approximately −3.68 ± 1.62 mV (Supplementary Figure S6). The neutral and negative particle particles and the inter-tissue have weak static interactions, so HA@ICG NPs have a greater capacity to flow into lymphatic vessels (Swartz, 2001; Ryan et al., 2014; Peng et al., 2021).

As shown in Figure 2E, the fluorescent emission peak of HA@ICG NPs is redshifted by approximately 15 nm in comparison to ICG. To further demonstrate the successful synthesis of nanoparticles, proton nuclear magnetic resonance (^1^H NMR) spectra and FTIR spectra were employed, as illustrated in Supplementary Figures S7–S10. All the above demonstrates successful self-assembly of HA@ICG nanoparticles. To verify the *in vitro* photostability of HA@ICG NPs, we continuously irradiated HA@ICG NPs and free ICG with an 808 nm laser and evaluated the fluorescence intensity changes of free ICG and HA@ICG NPs at different time points under a 1,000 nm long-pass filter using the InGaAs camera platform. The fluorescence of HA@ICG NPs decreased very gradually and stabilized later as exposure time increased (Figure 2F). The gentle decrease in fluorescence of HA@ICG NPs suggests that they are more stable and have better photostability *in vitro*. This characteristic creates further opportunities for the effective use of self-assembled nanoparticles for *in vivo* long-lasting fluorescence imaging.

### 3.2 Cytotoxicity assessment and cellular uptake ability of HA@ICG NPs *in vitro*

Since nanoparticles for bioimaging must be free of toxicity or side effects *in vivo*, the cytotoxicity of HA@ICG NPs on HLECs was tested in this study using the MTT assay (Figure 2G). The cell viability of the HLECs was maintained at more than 90% even when the concentration of ICG reached as high as 25 mM. The above results indicate that HA@ICG NPs have good biocompatibility and provide a safe basis for subsequent related experiments.

**FIGURE 3:**
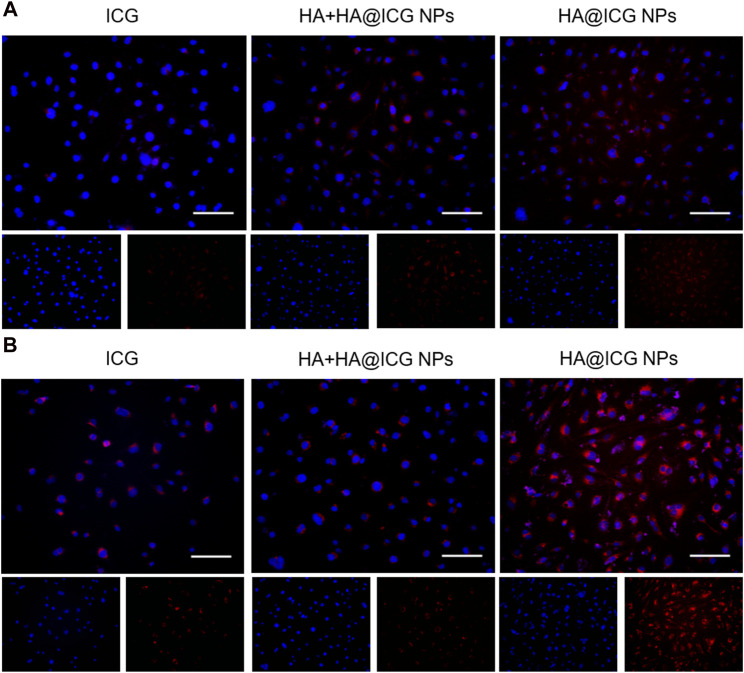
Images of HLECs incubated with different nanoparticle groups respectively for **(A)** 2 h and **(B)** 6 h. Scale bar = 100 µm. HA+HA@ICG NPs Group: HA (10 mg mL^−1^) pre-treatment for 1 h before adding HA@ICG NPs.

The cellular uptake ability of HA@ICG NPs was subsequently evaluated by coincubating HLECs with different nanoparticle groups for 2 h and 6 h (Figure 3). The fluorescence intensity exhibited a positive correlation with the duration of co-culture. To verify the critical role of HA molecules in the cellular uptake of nanoparticles, excess HA molecules (10 mg/mL) was previously co-cultured with the cells for 1 h. The results indicate that the group treated with HA@ICG NPs had the most intense red fluorescence, but the red fluorescence of the group pre-treated with HA was noticeably diminished. The competition between HA molecules and LYVE-1 for binding to the surface of HLECs, which hinders the cellular uptake of HA@ICG NPs, could explain this. The results verified that cells effectively internalised HA@ICG NPs by interaction with LYVE-1 on HLECs, which was consistent with the previous research (Yang et al., 2017).

### 3.3 NIR-II bioimaging of lymph vessels, lymph nodes, and blood vessels *in vivo*

**FIGURE 4:**
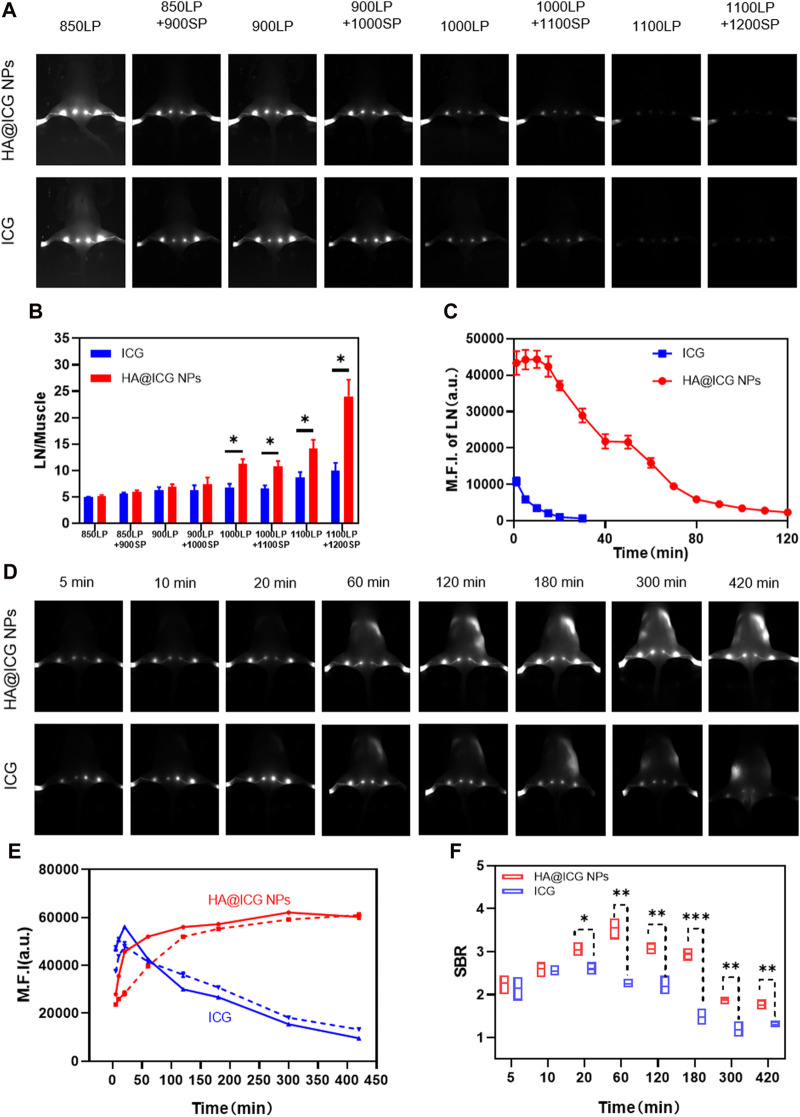
**(A)** Fluorescence images of popliteal and sacral LNs at different NIR long pass filters of HA@ICG NPs and free ICG. **(B)** The ratio of fluorescence intensity of LN to muscle at different wavelengths. **(C)** Fluorescence changes of the mouse hindlimb limb lymphatic system under continuous laser radiation over a period of time. **(D)** NIR-II fluorescence imaging of LNs and lymphatic vessels at different time points after s.c. injection of HA@ICG NPs and free ICG, respectively. **(E)** Time dependent fluorescence intensity of popliteal (solid line) and sacral (imaginal line) LNs. **(F)** A comparison of the signal-to-background ratio between the HA@ICG NPs group and free ICG for lymphatic vessel imaging. Data expressed as Mean ± SD (*n* = 3). *p < 0.05, **p < 0.01, and ***p < 0.001.

The lymphatic system is crucial in the human body, carrying out various important actions that are necessary for maintaining general health. Any malfunction in this system has the potential to result in the formation of diseases in several organs. Hence, it is essential to monitor and depict the lymphatic vessels and nodes to gain a comprehensive understanding of the drainage function of the lymphatic system, avert the occurrence of lymphatic diseases, and assess any associated pathologies that may emerge (Petrova and Koh, 2020; Xu et al., 2021). To investigate the NIR-II fluorescence imaging effect of HA@ICG NPs in lymph nodes and lymphatic vessels, we first searched for the optimal NIR-II imaging window for HA@ICG NPs. The mice were positioned in a prone position and injected with HA@ICG NPs and ICG through the foot pads at an iso-ICG concentration (300 µM). Fluorescence images of the lymphatic structures of the hindlimb were taken using an InGaAs camera under long pass filters of different wavelengths (Figures 4A, B). It is not difficult to find that there are problems such as autofluorescence interference of biological tissues, blurred boundaries, and poor resolution, although fluorescence signals of popliteal lymph nodes, sacra lymph nodes, and lymphatic vessels are displayed in the NIR-I window. However, when extended to the NIR-II region, the image resolution increased because of a decrease in autofluorescence interference and light scattering. As the wavelength increased from 850 nm to 1,200 nm, the fluorescence intensity ratio between lymph nodes and surrounding muscle tissues increased, and the image quality improved. However, this improvement came at the expense of losing the fluorescence signal. In summary, we selected 1,000 nm as the optimal NIR-II window for lymphatic imaging in this study.

In order to provide more evidence for nanoparticles to achieve clinical translation, this study firstly explores the photostability of HA@ICG nanoparticles *in vivo* after sustained laser irradiation (Figure 4C; Supplementary Figure S11). After 30 min of continuous irradiation, it was easy to see that the ICG group had nearly totally quenched; nevertheless, the nanoparticle group’s fluorescence signal may persist for up to 2 h. Therefore, it appears that the nanoparticles exhibit excellent photostability within a living organism, enabling the operator to have a sufficiently lengthy duration for performing tasks. Next, the *in vivo* photostability under non-continuous laser irradiation was investigated. After subcutaneous injection, HA@ICG NPs gradually increased and enabled prolonged visualization of lymph nodes for up to 7 h, while the fluorescence signal of ICG became significantly diminished after only 20 min (Figures 4D, E). This approach allows for more time for lymph node clearance and ensures complete clearance. Additionally, it allows for multiple lymphatic images, eliminating the inconvenience of repetitive injections. We also evaluated the image resolution of HA@ICG NPs for imaging slender lymphatic vessels. It was not difficult to find that the signal-to-background ratio of HA@ICG NPs group was always significantly higher than that of the free ICG group from 20 min onwards, indicating that the fluorescence intensity and image resolution of HA@ICG NPs were superior to those of free ICG group (Figure 4F). All of these features demonstrate that HA@ICG NPs have the potential to be translated to the clinic.

**FIGURE 5:**
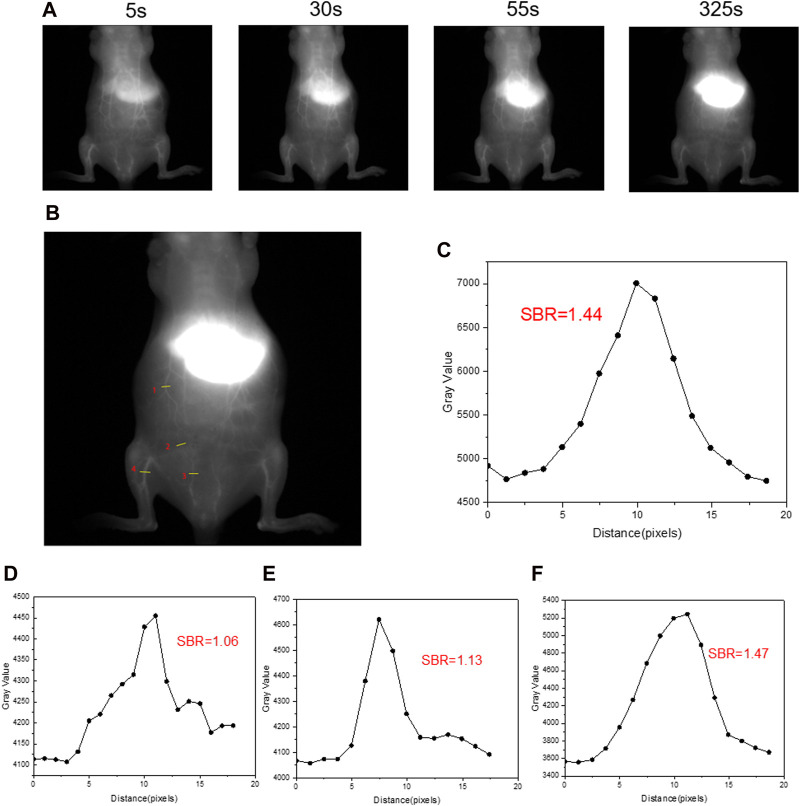
**(A)**The NIR-II vascular imaging in normal mice after intravenous injection under a 1,000 nm long-pass filter at different times. **(B)** ROI regions in NIR-II fluorescence images of blood vessels post i.v. injection of HA@ICG NPs at 325 s. **(C–F)** The signal-to-background ratio of four ROI regions (1, 2, 3, 4) in **(B)**. Injection dosage: 300 μM ICG, 150 μL; laser: 808 nm, 10 ms, 78 mW cm^−2^.

Further investigation into the vascular photography effect of HA@ICG NPs is recommended, as the above data suggest that it can have a favorable lymphatic system NIR-II imaging effect. The findings demonstrated that the mouse’s abdominal and peritoneal blood vessels were rapidly and clearly visible after receiving an intravenous injection of HA@ICG nanoparticles, and this display persisted for up to 5 min (Figure 5A). In addition, the quantitative analysis results showed image clarity characterised by a signal-to-background ratio of 1.47 (Figures 5B–F). Therefore, HA@ICG nanoparticles possess the capability of identifying minuscule blood vessels. Thus, utilising this nanoplatform enables surgeons to assess the micro-blood supply status in the operative region, potentially significantly decreasing the likelihood of excessive bleeding, and offering a vital foundation for clinical decision-making.

### 3.4 Biodistribution and long-term biosafety analysis

**FIGURE 6:**
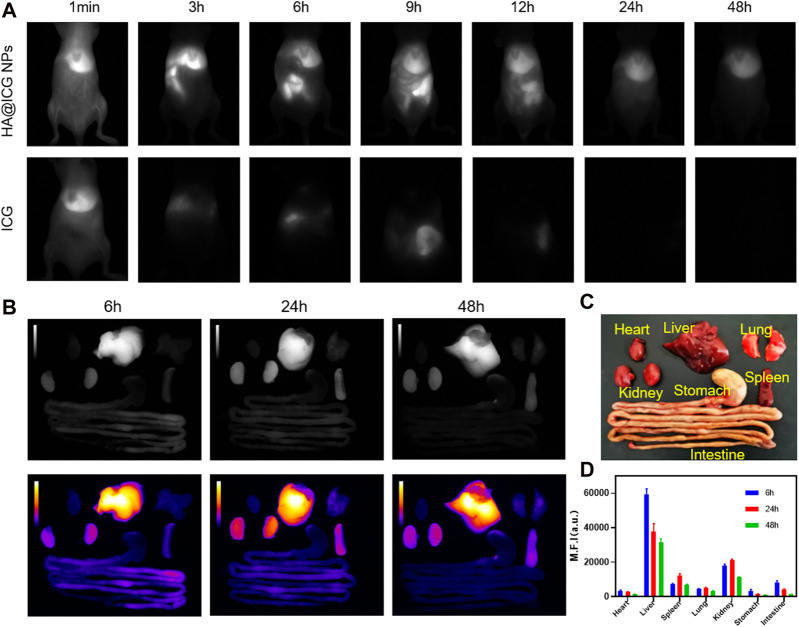
**(A)** Near-infrared II fluorescence images of mice at different time points after injection of HA@ICG NPs and ICG via caudal vein. **(B–D)** The NIR-II fluorescence imaging and quantitative analysis of isolated organs removed at 6, 24 and 48 h after tail vein injection of HA@ICG NPs.

Biodistribution and metabolism are key factors in ensuring the biosafety of nanomaterials and are one of the prerequisites for successful clinical translation. For this reason, whole-body NIR-II fluorescence images of mice were collected to evaluate the distribution of HA@ICG NPs and ICG at different time points post tail vein injection (Figure 6A). Then the major organs of mice injected with HA@ICG NPs were removed at 6, 24, and 48 h, respectively. NIR-II fluorescence imaging was performed on isolated organs and Image J software was used for quantitative analysis (Figures 6B–D). The fluorescence intensity was strongest in the liver, followed by the kidneys, both of which were significantly higher than the other organs, indicating that HA@ICG NPs are primarily metabolized by the liver and kidneys.

**FIGURE 7:**
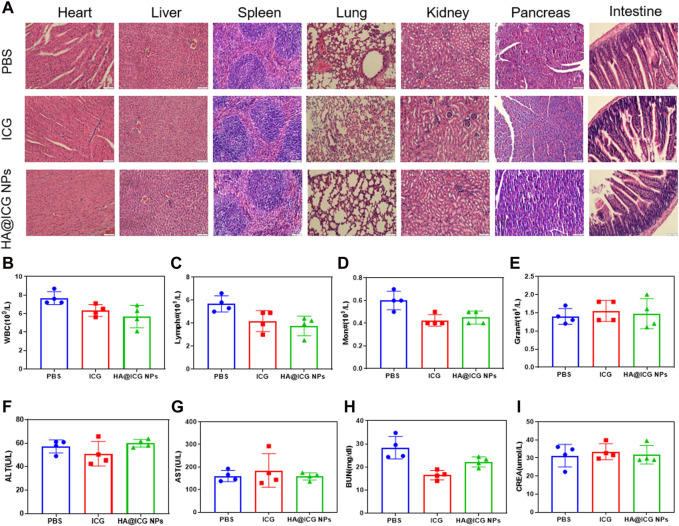
**(A)** H&E staining of the main organs from the mice including heart, liver, spleen, lung, kidney, pancreas and intestine after different treatments for 7 days. Scale bar = 100 μm. **(B–I)** Haemato-biochemical parameters of the mice after treatment with PBS, ICG, HA@ICG NPs for 7 days (*n* = 4).

Next we assessed the biocompatibility of HA@ICG NPs. First of all, Mice were sacrificed at 3 and 7 days post tail vein injection respectively and major organs were collected for H&E staining for histopathological studies. H&E staining illustrated no significant cell infiltration, deformation, necrosis and other pathological manifestations in major organs such as heart, liver, spleen, lung, kidney, pancreas, and intestine (Figure 7A; Supplementary Figure S12), either 3 or 7 days post injection. To further assess the long-term biosafety of HA@ICG NPs, we also performed blood routine and blood biochemistry analysis. Haemato-biochemical and blood routine parameters were within the normal ranges, including ALT, AST, BUN, CREA, WBC, RBC, PLT, and so on (Figures 7B–I; Supplementary Figures S13–S15). In conclusion, these results show that HA@ICG NPs has excellent biocompatibility *in vivo*, which is the primary requirement for the clinical translation of nanoprobes as imaging agents.

## 4 Conclusion

In summary, the development of biocompatible nanoplatforms for targeted biomedical imaging has been a hot research topic with great interest and commitment. Thus, biocompatible nanomaterials (HA@ICG NPs) were prepared by a copper-free click reaction based on the unique properties of the NIR-II fluorescence tail of indocyanine green (ICG). The rational design of the nanoparticle platform inhibited the π-π stacking interactions between ICG molecules and reduced the possibility of aggregated fluorescence bursts. Additionally, the ideal size of the nanoparticles and the modification of the surface HA, enhance the specificity of the image and reduce off-target incidents. It has also been verified that the nanoparticles have a sufficient imaging duration for the lymphatic system and a high level of image resolution in the NIR-II window, and the HA@ICG NPs are promising as an angiographic agent for assessing local microcirculation. *In vitro* and *in vivo* studies have demonstrated that the HA@ICG nanoplatform has excellent photostability and biosafety, as well as superior visualization capabilities of the lymphatic system, making it promising for future clinical applications. However, it is noteworthy that HA@ICG NPs have not yet been validated in disease models pertinent to the lymphatic system and encounters inherent constraints in the imaging of deep-seated lymphatic tissues. Therefore, we will further conduct a deeper and more comprehensive exploration in the future work.

## Data Availability

The original contributions presented in the study are included in the article/Supplementary Material, further inquiries can be directed to the corresponding authors.
