# Supplementary material for: Hyaluronic acid modified indocyanine green nanoparticles: a novel targeted strategy for NIR-II fluorescence lymphatic imaging
Source: Front Chem. 2024 Jul 3;12:1435627. doi: 10.3389/fchem.2024.1435627 (PMC11251975; doi:10.3389/fchem.2024.1435627)
Supplement: Supplementary file 1 [file DataSheet1.docx]

**Supporting Information**

**
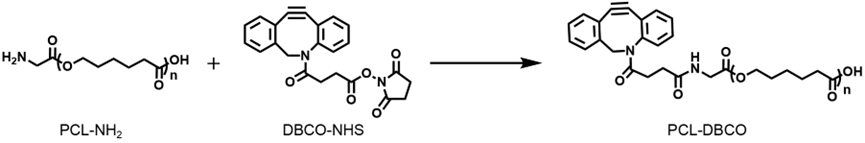
**

**Figure S1** Synthesis of compound PCL-DBCO

**
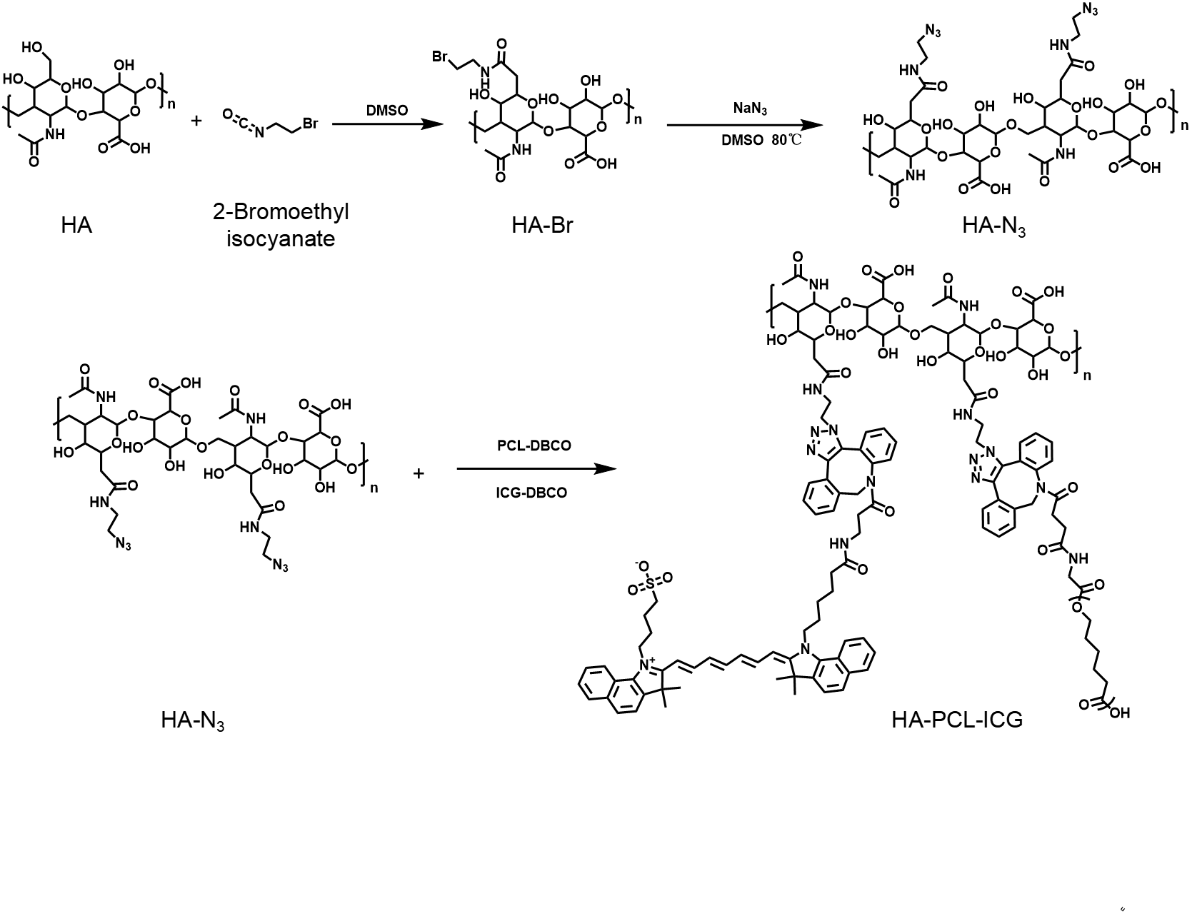
**

**Figure S2** Synthesis of compound HA-N_3_


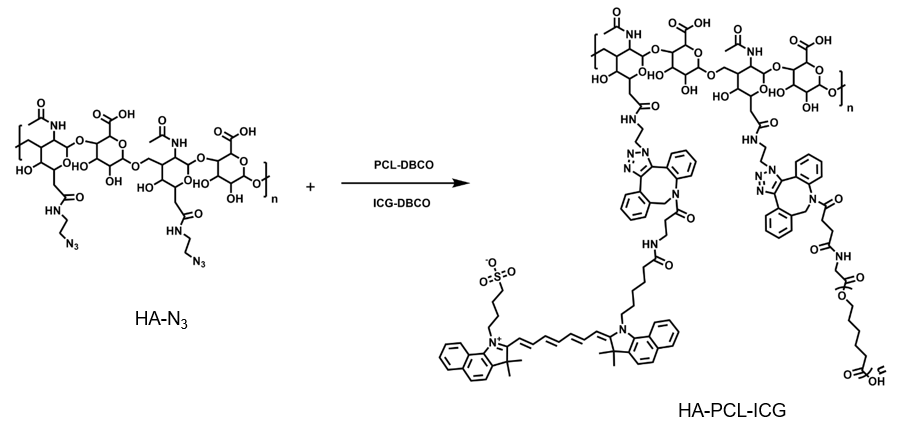


**Figure S3** Synthesis of compound HA-PCL-ICG


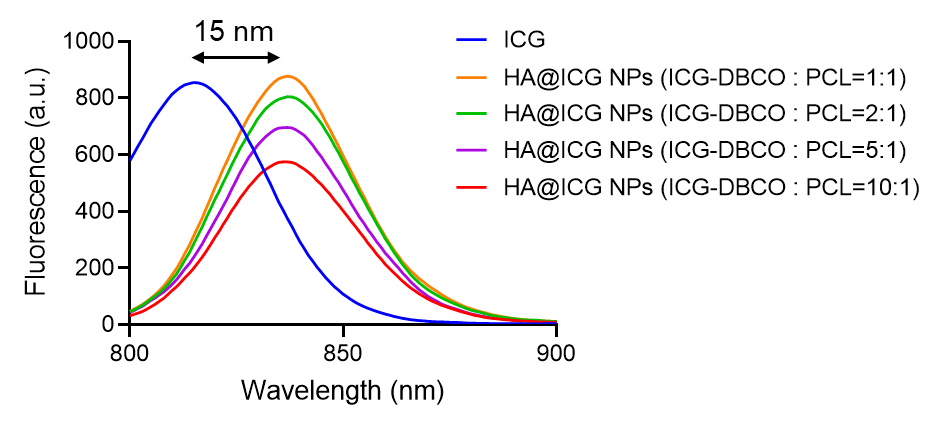


**Figure S4** The fluorescence spectrum of ICG and HA@ICG NPs with different ICG-DBCO to PCL ratios.


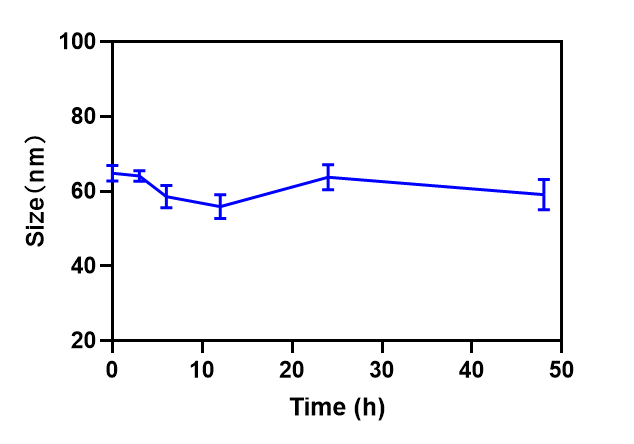


**Figure S5** DLS changes of HA@ICG NPs with cell culture medium containing 10% fetal bovine serum in 48 hours.


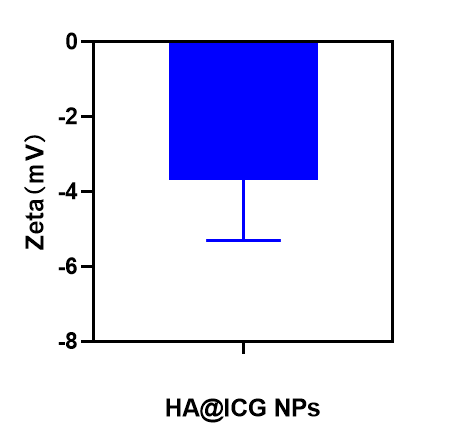


**Figure S6** Zeta potential of HA@ICG NPs


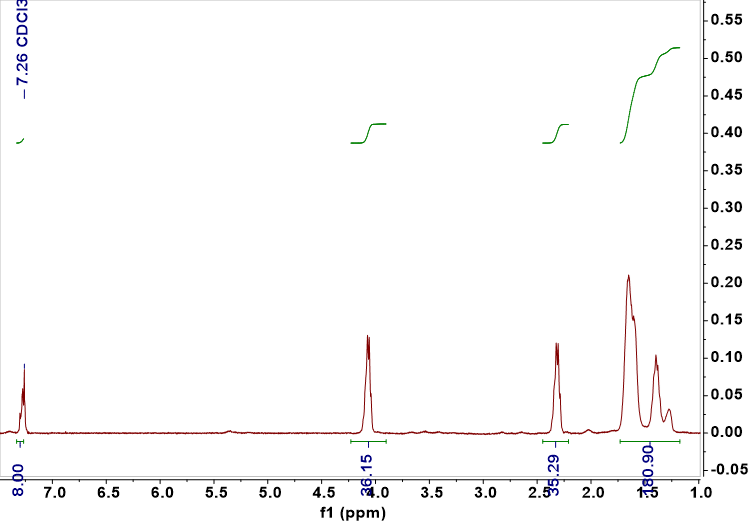


**Figure S7** ^1^H NMR spectrum (400 MHz, CDCl3, room temperature) of PCL-DBCO


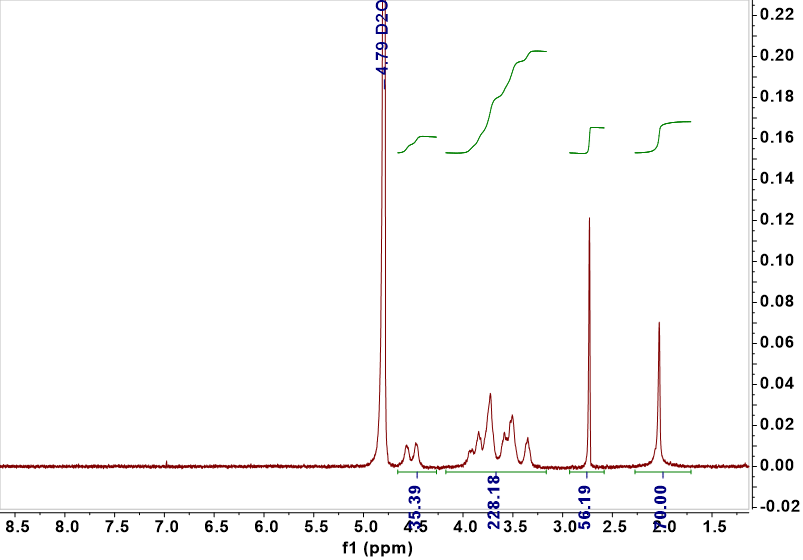


**Figure S8** ^1^H NMR spectrum (400 MHz, CDCl3, room temperature) of HA-N_3_


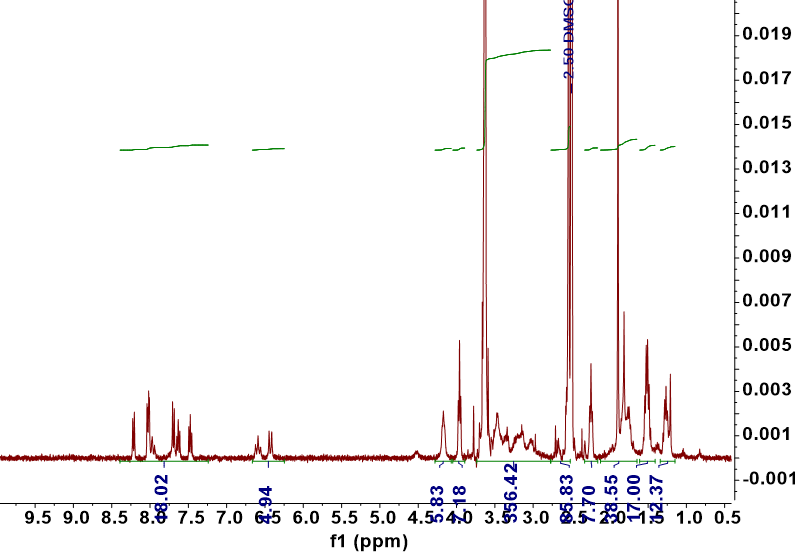


**Figure S9** ^1^H NMR spectrum (400 MHz, DMSO, room temperature) of HA-PCL-ICG


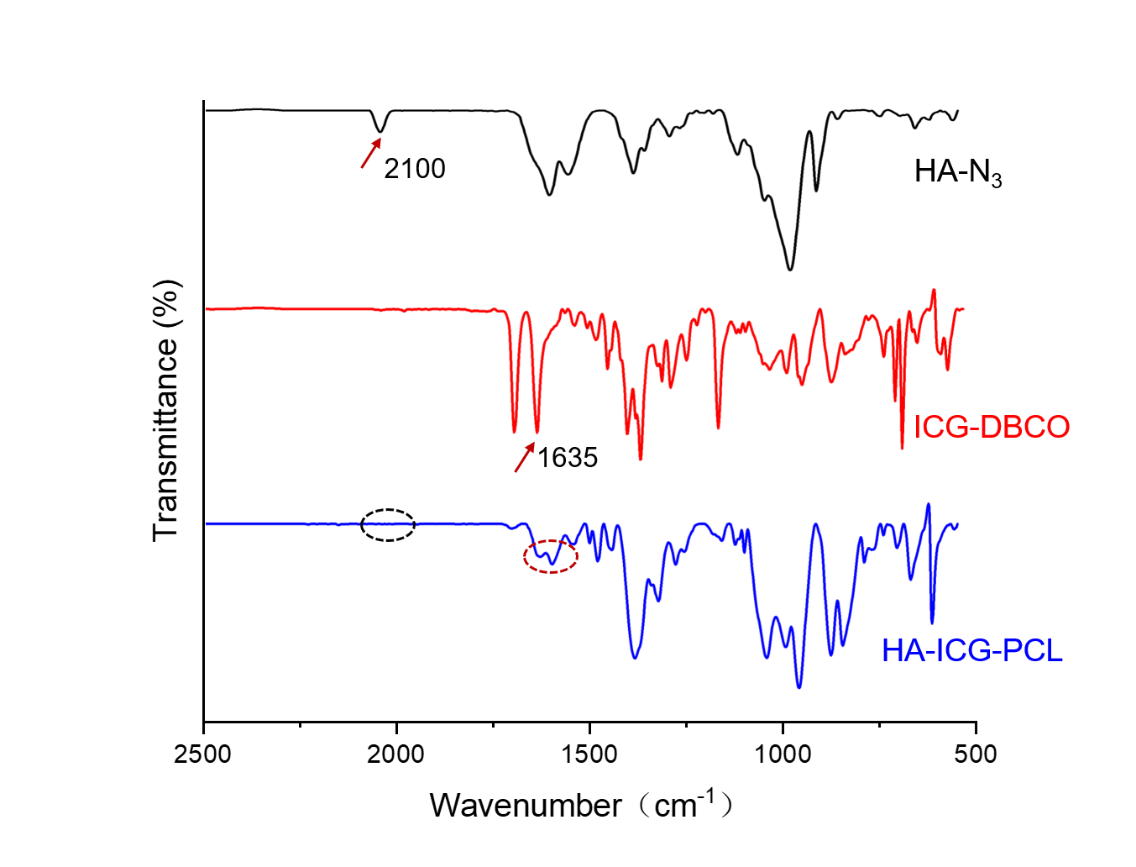


**Figure S10** FTIR spectroscopy of HA-N_3_, ICG-DBCO and HA-ICG-PCL, respectively.


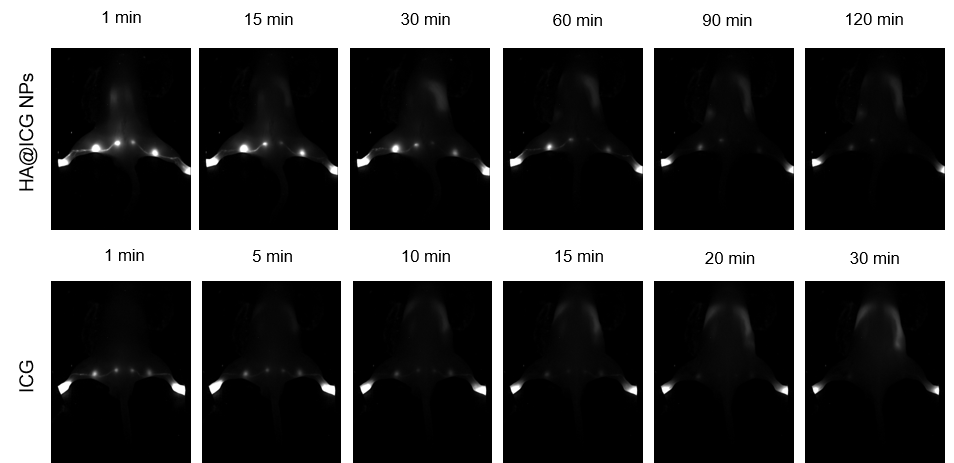


**Figure S11** NIR-II fluorescence imaging of the mouse hindlimb limb lymphatic system under continuous laser irradiation (808 nm, 78mW cm^−2^) over a period of time in a long-pass filter above 1000 nm.


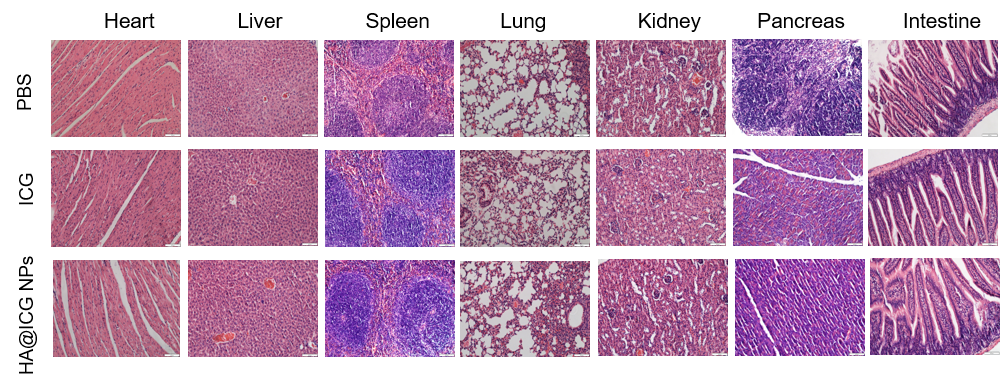


**Figure S12** H&E staining of the main organs from the mice including heart, liver, spleen, lung, kidney, pancreas and intestine after treatment with PBS, ICG, HA@ICG NPs for three days. Scale bar is 100 μm.

**
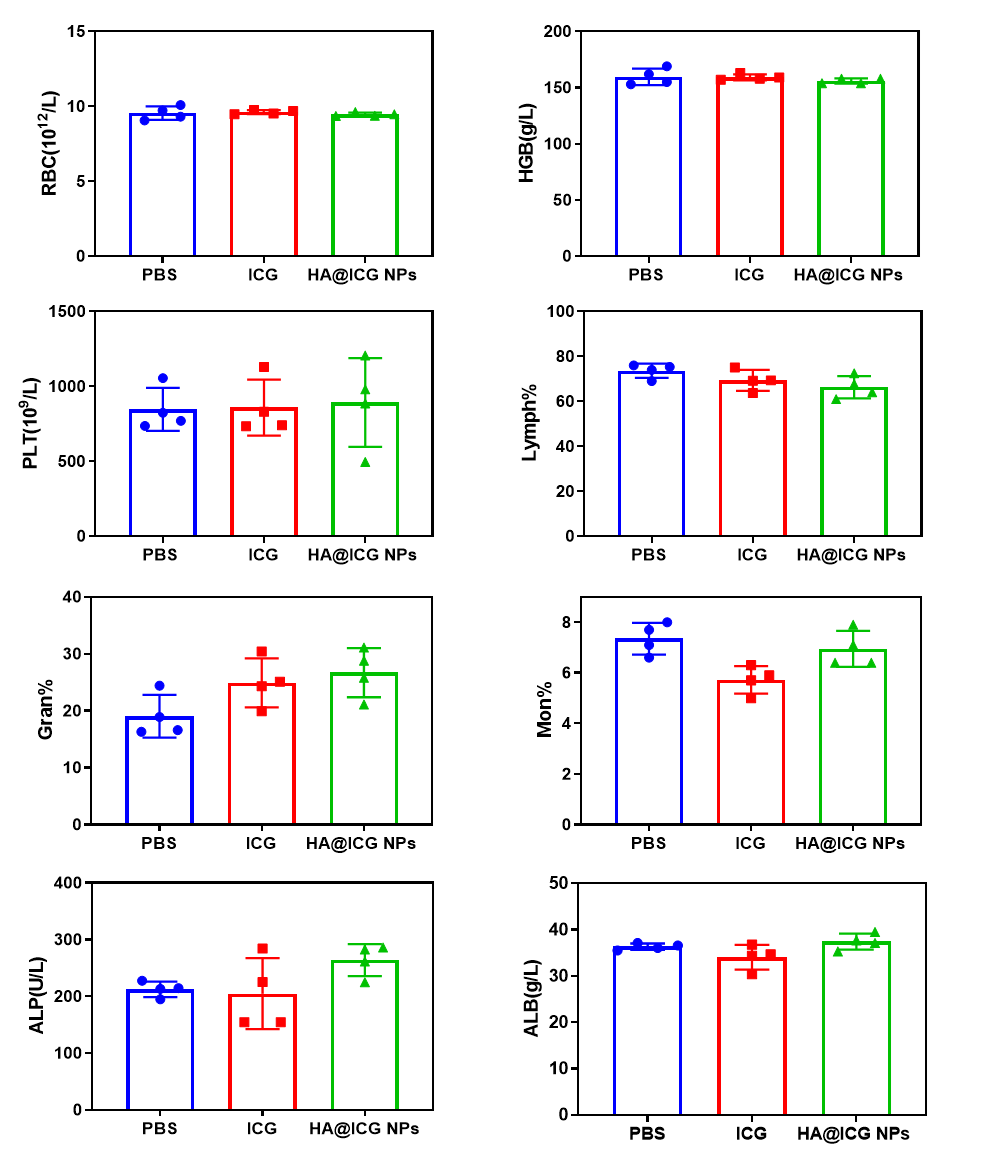
**

**Figure S13** Haemato-biochemical and blood routine parameters of the mice after treatment with PBS, ICG, HA@ICG NPs for seven days. n=4

**
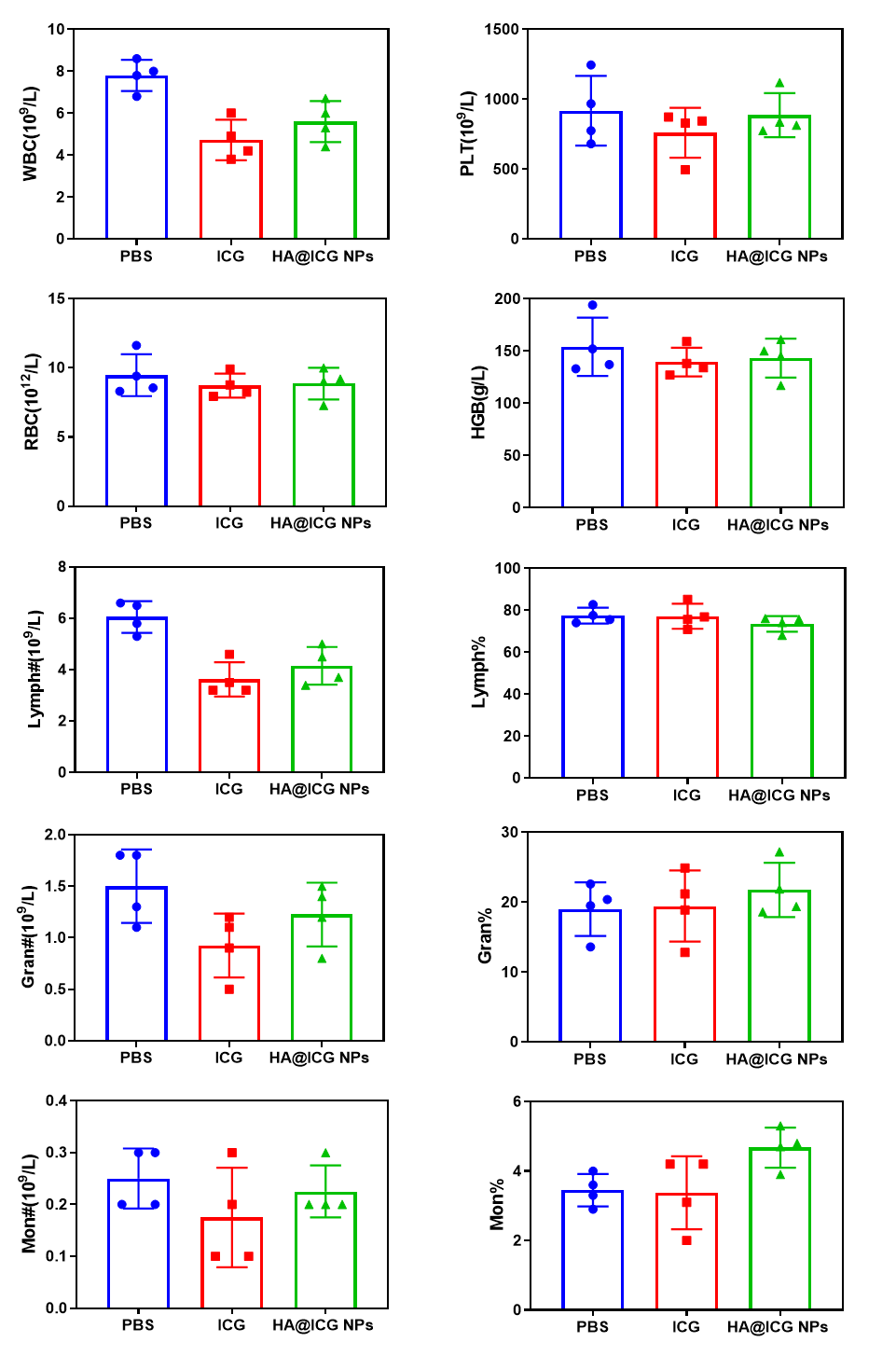
**

**Figure S14** Blood routine parameters of the mice after treatment with PBS, ICG, HA@ICG NPs for three days. n=4

**
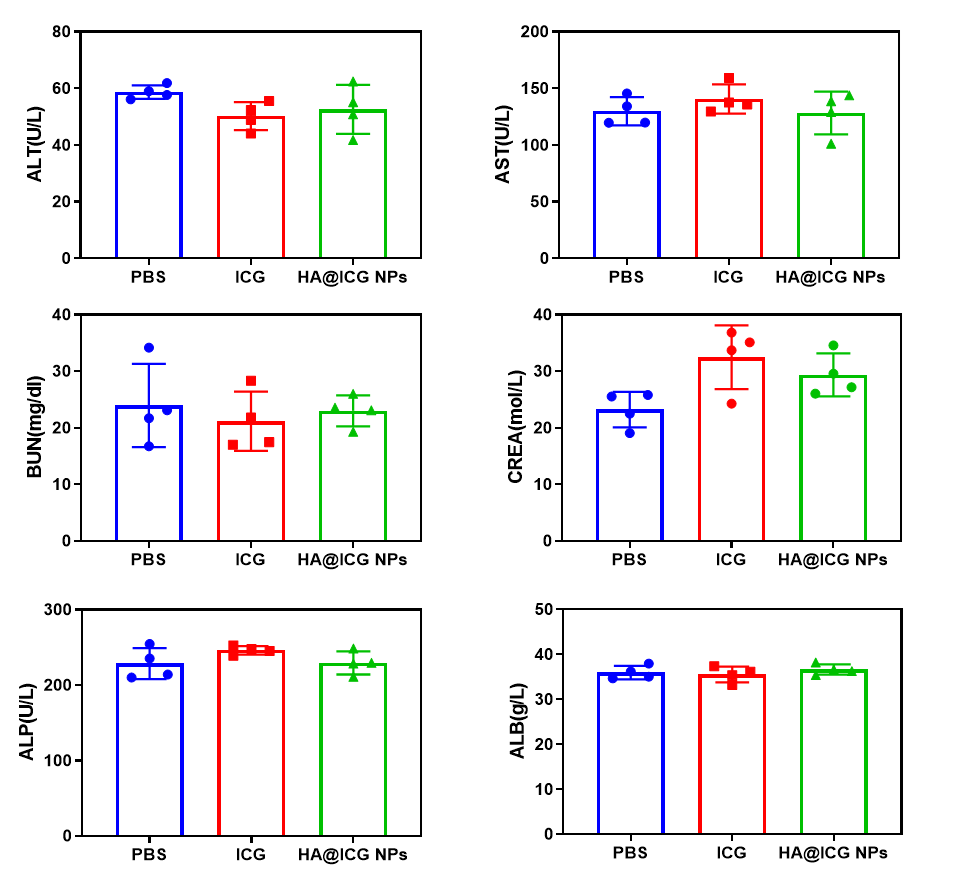
**

**Figure S15** Haemato-biochemical parameters of the mice after treatment with PBS, ICG, HA@ICG NPs for three days. n=4
